# Fertilization mode differentially impacts the evolution of vertebrate sperm components

**DOI:** 10.1038/s41467-022-34609-7

**Published:** 2022-11-10

**Authors:** Ariel F. Kahrl, Rhonda R. Snook, John L. Fitzpatrick

**Affiliations:** 1 Department of Zoology, Stockholm University, Svante Arrhenius väg 18B, SE-10691 Stockholm, Sweden; 2 Department of Biology, Hamilton College, 198 College Hill Road, Clinton, NY USA

**Keywords:** Sexual selection, Phylogenetics

## Abstract

Environmental change frequently drives morphological diversification, including at the cellular level. Transitions in the environment where fertilization occurs (i.e., fertilization mode) are hypothesized to be a driver of the extreme diversity in sperm morphology observed in animals. Yet how fertilization mode impacts the evolution of sperm components—head, midpiece, and flagellum—each with different functional roles that must act as an integrated unit remains unclear. Here, we test this hypothesis by examining the evolution of sperm component lengths across 1103 species of vertebrates varying in fertilization mode (external vs. internal fertilization). Sperm component length is explained in part by fertilization mode across vertebrates, but how fertilization mode influences sperm evolution varies among sperm components and vertebrate clades. We also identify evolutionary responses not influenced by fertilization mode: midpieces evolve rapidly in both external and internal fertilizers. Fertilization mode thus influences vertebrate sperm evolution through complex component- and clade-specific evolutionary responses.

## Abstract (web summary)

The location where fertilization takes place can be highly variable across species, and especially between internal and external fertilizers. Kahrl et al. find that fertilization environment plays a significant role in the evolution and diversification of sperm morphology across vertebrate species.

## Introduction

Understanding the selective forces that drive trait evolution among lineages is a central goal of evolutionary ecology. Transitions between ecological niches, along with shifts in ecological opportunity and competition, commonly drive morphological diversification^1–5^. Yet while evolutionary responses to novel environments are usually studied at the organismal level, the same concepts apply at the cellular level. Gametes experience a wide range of complex environments that can influence their morphological evolution^6–14^. Egg size among insects, for example, is influenced by oviposition ecology, with smaller eggs associated with aquatic oviposition and larger eggs associated with oviposition within an animal host^6^. The other gamete, sperm, exhibit tremendous variation in total length that is shaped by the postcopulatory sexually selected processes of sperm competition and cryptic female choice^7–9^. However, environmental factors can also exert selection on sperm morphology and influence how postcopulatory sexual selection operates^10–12^, thereby contributing to sperm evolutionary dynamics^13,15^.

The location of sperm deposition and/or where fertilization occurs (i.e., fertilization mode) represent major environmental factors that can alter how selection operates on gametes. For example, convergent changes in sperm size and morphology are observed among flatworms of the genus *Macrostomum* following transitions from copulations, where sperm are deposited inside the female reproductive tract, to hypodermic inseminations, where sperm are injected through the female’s epidermis and bypass the female reproductive tract^15,16^. Similarly, whether fertilization takes place outside of (external fertilization) or within (internal fertilization) the female can profoundly impact sperm morphology and evolution^14^. Recently, Kahrl et al.^13^ showed that fertilization mode across the animal tree of life impacts the evolution of total sperm length. Internal fertilizers are characterized by longer sperm that evolve faster with more extreme shifts in length compared to external fertilizers^13^. Such differences in evolutionary rates of diversification are commonly interpreted as arising from differences in the strength or form of selection acting on traits^17–21^. Kahrl et al.^13^ argued that the faster rate of total sperm length diversification in internal fertilizers is likely driven by shifts in the strength and mechanisms of postcopulatory sexually selected processes that vary with fertilization mode across species. While growing empirical evidence points to environmental factors impacting sperm evolution, sperm are typically made of component parts (e.g., the head, midpiece, and flagellum) that collectively influence sperm function. However, to our knowledge, no broad phylogenetic study has addressed how the location of fertilization influences the evolution of individual sperm components.

Sperm are typically motile and charged with the task of finding and fertilizing an egg before sperm from rival males. This task is reflected in the function of the individual sperm components of the sperm cell. The sperm head houses the genetic material and acrosome (when present) and interacts with the egg’s cell membrane prior to fertilization, while the sperm midpiece houses mitochondria that supplies the energy required for the sperm flagellum to generate the propulsive force for sperm motility. Each component plays a distinct role in the function of the cell. Therefore, the morphology of individual sperm components may respond differently to selection based on the environment in which they operate^14,22^. Environments differ substantially between fertilization modes^23,24^. The internal fertilization environment is more viscous, for example, which can influence the dynamics and kinematics of sperm swimming performance^25^. Fertilization mode is also related to the duration of sperm longevity. Sperm from external fertilizing species typically remaining functional for seconds, minutes, or hours after they are released^26–29^, whereas sperm of internal fertilizers can remain functional within the female reproductive tract for days, months, or even years^30,31^. Such differences in fertilization environments and sperm performance have the potential to differentially target sperm components between fertilization modes.

Yet, whether fertilization mode impacts sperm component length evolution remains unknown. An early observation (without any formal analysis) suggested that sperm components are longer in some internal fertilizing marine invertebrates compared to external fertilizing species^14^. Two recent empirical studies suggest that the impact of fertilization mode on individual sperm components may be less straight-forward, finding inconsistent effects of fertilization mode on sperm component length evolution^32,33^. However, these studies had a narrow taxonomic focus, examining 10 or fewer species^32,33^. Thus, whether fertilization mode impacts the evolution of individual sperm components within larger clades or across broad taxonomic groups remains unclear.

Moreover, evolutionary responses in total sperm length need not be reflected in every sperm component. While the independent evolution of each sperm component may not be possible due to genetic constraints^34–36^, a handful of recent studies report that sperm components exhibit distinct evolutionary rates of phenotypic diversification in internally fertilizing birds, mammals, reptiles, and insects^18,19,37–40^. These findings suggest that sperm components can evolve independently. Consequently, whether fertilization mode consistently (or differentially) impacts the evolution of both the length and rate of sperm component diversity is unknown. Determining whether and how consistently fertilization modes influence sperm component evolution is critical to explain why, despite performing the same function of fertilizing eggs across animals, sperm exhibit such tremendous morphological diversity.

Here, we test the hypothesis that fertilization mode influences evolutionary patterns and rates of sperm component lengths in vertebrates. Vertebrates represent an exceptional model to study how fertilization mode shapes sperm morphological evolution as there is ample data available on the morphology of individual sperm components compared with other taxa^41^, they exhibit variation in fertilization modes (i.e., external and internal fertilization) both among and within major vertebrate clades^13^, and the well-resolved vertebrate tree facilitates examination at a broad taxonomic scale. We take advantage of these attributes to contrast patterns of evolutionary divergence of sperm component lengths between fertilization modes and vertebrate classes and to compare rates of evolution among sperm components.

We show that fertilization environment influences both the lengths and rates of evolution of the sperm components. However, the general patterns observed across species become more variable within vertebrate clades. This suggests that while fertilization mode plays an important role in the evolution of the sperm components, complex patterns of selection that exist within clades also shape the evolutionary divergence of sperm components in vertebrates.

## Results

### Fertilization mode influences sperm component lengths

**Fig. 1:**
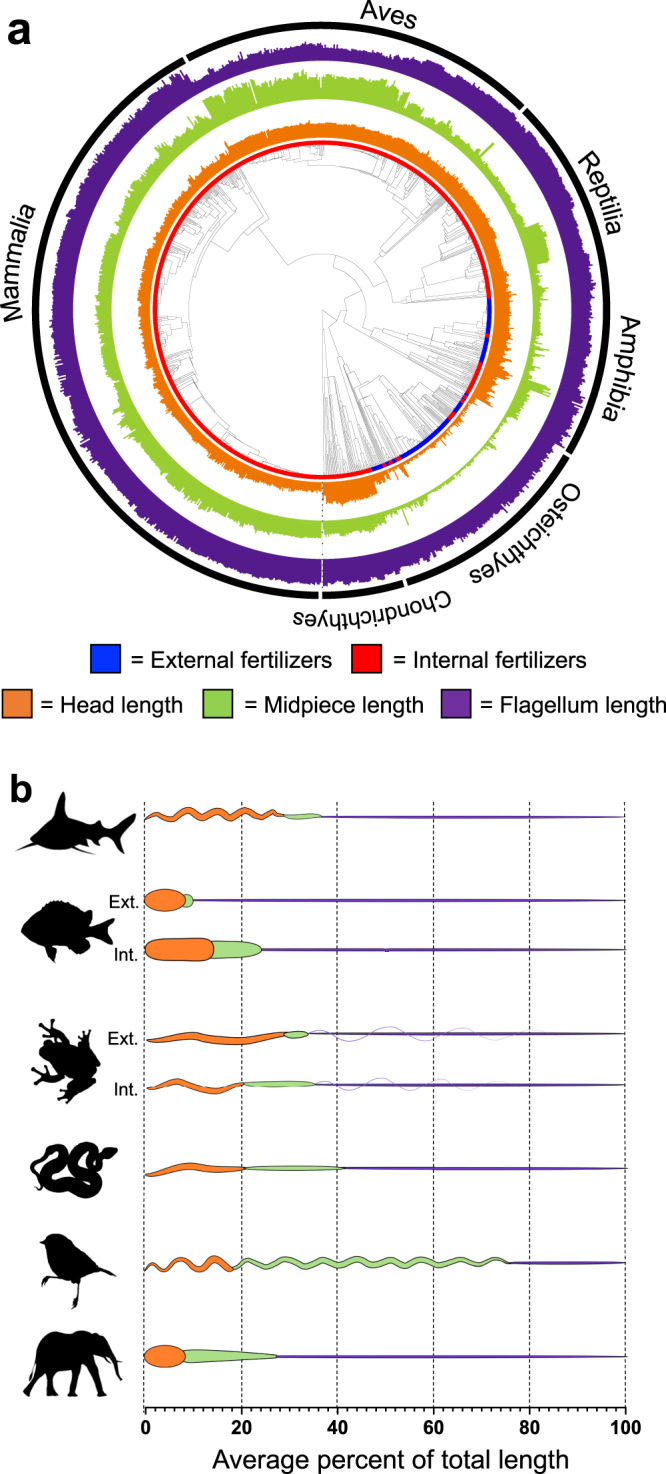
Sperm morphology across vertebrates. **a** The phylogeny of the species in our dataset where sperm data are available. Sperm head (orange), midpiece (green), and flagellum (purple) length (plotted as natural log-transformed values) varied within and among vertebrate clades. Colors on the tips of the tree indicate whether the species is an external fertilizer (blue) or an internal fertilizer (red). Data were available from six vertebrate clades (i.e., superclass/class), including Chondrichthyes (*n* = 51), Osteichthyes (*n* = 134), Amphibia (*n* = 104), Reptilia (*n* = 117), Aves (*n* = 237) and Mammalia (*n* = 460, Table S1). **b** Sperm morphology varied among major vertebrate clades. The average percent of each sperm component for each vertebrate clade is presented for (from top to bottom) Chondrichthyes, Osteichthyes, Amphibia, Reptilia, Aves, and Mammalia. For Osteichthyes and Amphibia, average sperm morphologies are presented separately for external (Ext.) and internal (Int.) fertilizing species. As expected, there were clear differences in the lengths of different sperm components across vertebrates: sperm head was shortest, followed by midpiece, and the flagellum was longest on average (Table S1). This general pattern (i.e., average sperm head <midpiece <flagellum length) was also found among Mammalia and Reptilia (Table S1). However, a different pattern was observed in other vertebrate clades. The sperm head was longer on average than the midpiece in Chondrichthyes, Osteichthyes, and Amphibia, while in Aves the sperm midpiece was longer on average than either the sperm head or flagellum (Table S1). The stylized sperm drawings represent common sperm morphologies in each group and are used to illustrate broad similarities and differences among vertebrate clades. However, we acknowledge that there is wide variation in sperm morphologies within these vertebrate clades that is not depicted here. Silhouette illustrations contributed by various authors under public domain license (CC0 1.0 license) from PhyloPic (http://phylopic.org). Source data are provided as a source data file.

Data on sperm head, midpiece, and flagellum length were collected from 1103 vertebrate species the SpermTree data repository^41^, including 191 external fertilizers and 912 internal fertilizers from six vertebrate clades (Fig. 1a and Table S1). Importantly, two vertebrate superclass/classes—the Osteichthyes and Amphibia—exhibit variation in fertilization mode, containing both external and internal fertilizing species, facilitating within-clade analyses (Table S1). Fertilization modes for all species in the dataset came from assignments specified in Kahrl et al.^13^. Ancestral state reconstructions among vertebrates in our dataset revealed ~13 independent transitions from external fertilization to internal fertilization and no back transitions across the vertebrates considered in our analyses (Table S2). Among Osteichthyes and Amphibia, there were 6 and 3 independent transitions from external to internal fertilization.

**Fig. 2:**
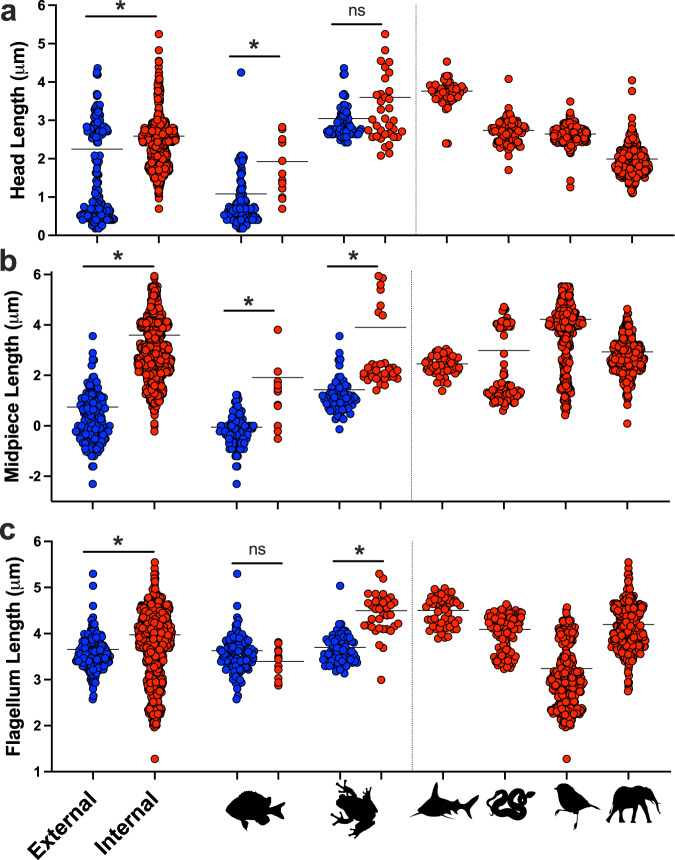
Sperm length for internally and externally fertilizing vertebrates. Sperm length (on natural log scale) of the sperm head (**a**), midpiece (**b**), and flagellum (**c**) for external (blue) and internal fertilizers (red), plotted with the mean of each group. Plotted are values for all external and internal fertilizers in our dataset, and each of the major vertebrate classes: (from left to right) Osteichthyes, Amphibia, Chondrichthyes, Reptilia, Aves, and Mammalia. Osteichthyes and Amphibia are broken into the external and internal fertilizing species. Significant differences in sperm component lengths between fertilization modes are provided for comparisons between externally fertilizing vertebrates (*N* = 1103), fish (*N* = 134), and amphibians (*N* = 104) which were calculated using phylogenetic linear models (* = *P* < 0.05, see Table S3 for statistical model outputs for each comparison). Silhouette illustrations contributed by various authors under public domain license (CC0 1.0 license) from PhyloPic (http://phylopic.org). Source data are provided as a Source Data file.

Phylogenetic linear models revealed that sperm component lengths varied within and between vertebrate clades (Fig. 1 and Table S3). Despite this variation, we found a consistent evolutionary signal of fertilization mode on sperm morphology across vertebrates. All sperm components were significantly longer in internal than in external fertilizers across vertebrates (Fig. 2). Internally fertilizing species had sperm heads that were on average 1.4× longer (Table S1 and Fig. 2a), midpieces that were 18× longer (Table S1 and Fig. 2b) and flagella that were 1.3× longer (Table S1 and Fig. 2c) than external fertilizers across vertebrates.

However, in the two clades that exhibit variation in fertilization mode (Osteichthyes and Amphibia), effects of fertilization mode on sperm length components differed both from the pattern across vertebrates and from each other. While the head (Fig. 2a) and midpiece (Fig. 2b) were longer in internal fertilizing Osteichthyes, flagella length did not differ between fertilization modes (Table S3 and Fig. 2c). In contrast, in Amphibia, the midpiece (Fig. 2b) and flagellum (Fig. 2c) were longer in internal fertilizing species, but sperm head length did not differ between fertilization modes (Table S3 and Fig. 2a).

### Fertilization mode has variable effects on rates of sperm component length evolution

We tested for differences in evolutionary rates of each sperm component length between fertilization modes using the ‘*OUwie’* function from the R package *OUwie* v. 2.1^42^. Fertilization mode influences the rate of evolution of sperm component lengths and this influence varied across sperm components and across the taxonomic level of analysis. Fertilization mode influenced the rate of evolution for both head and flagellum lengths (OUMVA and OUMV models supported, respectively; Table S4a) but in different directions. Flagella length evolved 2× faster in internal fertilizers (σ^2^ = 0.047 ± 0.0001) than external fertilizers (σ^2^ = 0.024 ± 0.00007, *t* = 867.42, df = 49, *P* < 0.001; Table S4a) but head lengths evolved almost 2× faster in external fertilizers (σ^2^ = 0.013 ± 0.0005) than in internal fertilizers (σ^2^ = 0.007 ± 0.002, *t* = 5.83, df = 49, *P* < 0.001, Table S4a). In contrast to the other two components, evolutionary models supported an interpretation in which the rate of sperm midpiece length evolution is not impacted by fertilization mode (OUMA model supported; Table S4a).

These patterns differed when separately examining Osteichthyes and Amphibia, which also differed from each other. Osteichthyes had a completely opposite pattern to the wider vertebrate dataset: sperm head and flagellum length did not differ between fertilization modes (OUM; Table S4b), while sperm midpiece length evolved 12× faster in internal fertilizers than external fertilizers (OUMV model supported; Table S4b). In Amphibia, rates of evolution for all sperm components were higher in internal fertilizers (OUMVA, OUMVA, and OUMV models supported, respectively; Table S4c), with sperm head, midpiece and flagellum evolving 27×, 139× and ~2× faster, respectively, in internal compared to external fertilizers. This disparity in rates between internal and external fertilizing amphibians likely arises from clade-level differences, as there are relatively few shifts in fertilization mode among amphibians.

### The midpiece evolves rapidly relative to head and flagellum lengths

**Fig. 3:**
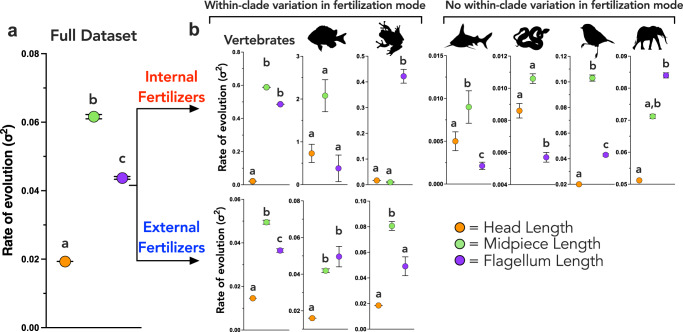
Rates of evolution of the sperm head, midpiece, and flagellum lengths. Rates of evolution were estimated using *mvMORPH* for all vertebrates in our dataset (Full dataset, **a**), external and internal fertilizers (divided under Vertebrates), and for each vertebrate class (from left to right) including Osteichthyes, Amphibia, Chondrichthyes, Reptilia, Aves, and Mammalia (**b**). Separate analyses for internal and externally fertilizing Osteichthyes and Amphibia are presented. Rates of evolution are presented for sperm head (orange), midpiece (green) and flagellum (purple) in all plots. Internal fertilizers are on the top row, and external fertilizers are on the bottom row. Mean and standard error estimates for each evolutionary rate were estimated using a boot-strapping approach with 100 bootstrap samples. Differences in the rates of evolution of sperm components were determined using post hoc tests that compared the rates of evolution in a pairwise manner using AICc to determine the model with the best fit (rates were equal, or rates were different, see details in “Methods”). When models with different rates had the lowest AICc value, rates were considered to be significantly difference from one another, which is indicated with letters above the estimated rate values. Silhouette illustrations contributed by various authors under public domain license (CC0 1.0 license) from PhyloPic (http://phylopic.org). Source data are provided as a Source Data file.

Above, we tested for the effect of fertilization mode on the evolutionary rate of each individual sperm component. Here we tested for differences in the rates of evolution among sperm components and determined whether there are consistent patterns between fertilization modes and across vertebrate clades using the function ‘*mvOU’* in the package *mvMORPH* 1.1.4^43^. We found that sperm midpiece length evolved ~3× faster than sperm head length and 1.4× faster than flagellum length, and flagellum length evolved 2.3x faster than sperm head length across vertebrates (Fig. 3; Table S5). However, this broad pattern (evolutionary rate: midpiece > flagellum > head) was not consistent across fertilization modes and between vertebrate clades.

For external fertilizing vertebrates, midpiece length evolved faster than flagellum length, which evolved faster than head length (Fig. 3; Table S5). For internal fertilizing vertebrates, the rate of midpiece length evolution was indistinguishable from flagellum length, but both evolved at faster rates than sperm head length. Within-clade analysis for both internal and external fertilizers generally supported the broad pattern that sperm midpiece evolved faster, or as fast as, all other sperm components (Fig. 3; Table S5). The exception to this general pattern is internally fertilizing Amphibians, in which flagellum length evolved faster than either midpiece or head length, the latter two evolving at comparable rates. Internally fertilizing Osteichthyes also represent an exception to this general pattern, where the rate of evolution of all sperm components were statistically indistinguishable from one another despite the rate of sperm midpiece evolution being higher than all other sperm components. However, it is not possible to determine whether the lack of statistical difference in evolutionary rates among sperm components in internally fertilizing Osteichthyes arises from biological reality or due to a lack of statistical power given limitations in data availability for internally fertilizing Osteichthyes (*n* = 13).

Sperm head length evolved slowest across vertebrates irrespective of fertilization mode (Fig. 3; Table S5). Analyses for each clade were broadly consistent with this pattern, except in Chondrichthyes, where sperm head evolved at an intermediate rate between flagellum and midpiece length, and Reptilia, where sperm head evolved faster than the flagellum and was not distinguishable from the rate of sperm midpiece length (Fig. 3; Table S5).

Sperm flagellum length exhibited the most variable pattern of evolution compared to other sperm components. Flagellum length evolved either at a slower rate than all other sperm components (Chondrichthyes, Reptilia), at an intermediate rate between sperm midpiece and head length (externally fertilizing Amphibia, Aves), at a rapid rate that is indistinguishable from sperm midpiece rate (externally fertilizing Osteichthyes, Mammalia), at a slow rate that is indistinguishable from sperm head length (internally fertilizing Osteichthyes), or at a rate faster than any other sperm component (internally fertilizing Amphibia) (Fig. 3; Table S5).

## Discussion

We provide clear evidence that shifts in fertilization mode influence the lengths and rates of evolutionary diversification in sperm components both across vertebrates and within vertebrate clades. Overall, an internal fertilization environment is associated with longer sperm heads, midpieces, and flagella, a pattern suspected for more than 60 years^14^. Yet, how fertilization mode influences sperm length and rates of evolution varied among sperm components and vertebrate clades. For example, patterns are more ambiguous when assessed in fishes and amphibians. These taxa have fewer described transitions between fertilization mode relative to the total number of transitions observed across vertebrates^44^, which may impact detected patterns. Regardless, our findings suggest that the sperm head, midpiece, and flagellum can exhibit distinct evolutionary trajectories and raises the possibility that sperm components may respond differentially to selection. Intriguingly, sperm length evolution is not completely explained by fertilization mode. In particular, sperm midpiece length typically evolved faster than other components across vertebrate clades, irrespective of fertilization mode. Thus, while our results suggest fertilization environment shapes the evolution of individual sperm components, akin to recent findings for total sperm length^13^, these findings also indicate that the effects of fertilization mode must be viewed through the prism of clade-specific fertilization environments across the vertebrate tree of life.

Differences in postcopulatory sexual selection linked with variation in fertilization environment likely represent a key agent of selection driving sperm component length evolution. The dilution of sperm in external fertilizers typically results in raffle-based sperm competition mechanisms favouring the evolution of many small sperm^13,45^. This may explain the reduced sperm component lengths in most external fertilizers. In contrast, the internal fertilization environment increases potential for displacement-based mechanisms of sperm competition. These mechanisms favor larger sperm at the cost of producing more sperm^13,46^. Among internal fertilizers, displacement mechanisms of sperm competition are more likely when female reproductive tracts are small^47–49^. Under these conditions, sperm competition is expected to generate selection for increases in sperm size in small bodied species and increases in sperm number in large bodied species^13,46,50,51^. The broad range in female body size among internally fertilizing vertebrates, and the corresponding variation in female reproductive tract dimensions^52^, likely influences which sperm competition mechanism predominates among internally fertilizing vertebrates^46^. The flagellum is proportionally the largest component of the cell in most clades (except Aves, see Fig. 1b). Assuming that producing sperm with longer flagella is costly^47^, flagella length, in particular, may be targeted by sperm size/number trade-offs due to dilution effects. This may explain why flagellum length is both longer and evolves faster in internal fertilizers where fertilization dynamics can span from raffle to displacement mechanisms of sperm competition.

Differences in the biophysical properties of fertilization environments and the dynamics of female-sperm interactions between internal and external fertilizers could also drive the overall elongation of sperm components in internal fertilizers. Viscosity^48,49^ and the heterogeneity of the fertilization environment^52,53^ are both greater in internal than external fertilizers. There is also more scope for female-sperm interactions in internal fertilizers^7,54,55^, potentially exerting selection to elongate sperm component lengths. Such female-sperm interactions typically take place in the female reproductive tract. For example, sperm length is smaller among traumatically inseminating flatworms, where sperm bypass the female reproductive tract, compared to copulating species^15^.

The elongation of sperm flagellum and head lengths in internal fertilizers could be also related to their predicted effects on sperm function. Longer flagella length in internal fertilizers (with the exception of fishes) may generate greater propulsive force when in a more viscous environment, like in internal fertilizing species^56^. Likewise, a more viscous environment could select for longer sperm head lengths in internal fertilizers (with the exception of Amphibians) for improved forward movement^57–60^. However, the intra-specific evidence for a relationship between head and flagella lengths and sperm performance is mixed^59–62^. Moreover, sperm operating in the female reproductive tract experience “wall effects” that impact sperm movement and interacts with drag and propulsion in complicated ways^59^. While examining sperm component traits in isolation may be overly simplistic (i.e., the sperm midpiece and flagellum typically share an axoneme and may jointly act to influence motility), overall performance-based explanations appear insufficient to explain the variation in sperm component lengths between fertilization modes.

The midpiece is the only sperm component that is consistently longer in internal compared with external fertilizers, regardless of the taxonomic level of analysis. Sperm with longer midpieces are expected to produce more adenosine triphosphate (ATP)^63,64^, influencing aspects of sperm performance (e.g., swimming speed or longevity). As sperm typically remain active longer in internal fertilizers^30,31^, selection may be particularly strong on the midpiece in these species. However, while midpieces were longer in internal fertilizing taxa, the rate of sperm midpiece length evolution did not differ between fertilization modes. When combined with the comparison of rates of evolution among sperm components, we argue that the similarity in the rate of sperm midpiece evolution between fertilization modes stems from strong selection on the midpiece in both of these groups. Across vertebrates and in most vertebrate clades, midpiece length consistently evolves either faster or as fast as other sperm components, varying only for internal fertilizing amphibians. Narrower taxon studies in internally fertilizing species (birds, mammals, reptiles, and insects) also demonstrate that the sperm midpiece commonly evolves faster than other sperm components^18,19,37–40^. This suggests that the sperm midpiece is under strong selection, regardless of fertilization mode.

However, determining how selection acts on midpiece length will require greater clarity on the links between midpiece length, energy production, and sperm longevity—none of which enjoy broad or consistent empirical support^65^. Specifically, whether the size of the midpiece is related to the amount of energy produced (both positive^66^ and negative^67^ relationships have been reported) and whether mitochondrial number or volume increases with midpiece length^66,68^ are key outstanding questions. Moreover, whether sperm with longer flagella require more energy, selecting for longer midpieces, and how metabolic activity in the midpiece interacts with the metabolic activity in the flagellum remains unclear^66^. Clarifying the links between sperm energetics, morphology, and performance across a broad range of internal and external fertilizers is an important next step.

Both biophysical effects and interactions with the egg could influence sperm head length evolution. Despite sperm head length being smaller on average in external fertilizers, the rate of sperm head length evolution was faster in external fertilizers than internal fertilizers, with the exception of Amphibians. In amphibians, external fertilization often takes place in the context of a viscous fertilization environment (e.g., foam nests or terrestrial fertilization), which could explain why head length did not differ between external and internally fertilizing Amphibia. In contrast, sperm head length was small in externally fertilizing Osteichthyes, where the effects of viscosity are reduced. The sperm head also represents a point of contact with the egg surface, potentially generating co-evolutionary dynamics between the sperm and egg morphology^69,70^. The extent to which co-evolutionary dynamics between sperm head length and egg morphology differ between fertilization modes and differentially impact the evolution of sperm head length remains to be determined.

In conclusion, we provide a broad, phylogenetically-controlled, analysis on the long-standing hypothesis that fertilization mode shapes sperm component length across vertebrates. We find that environmental shifts in fertilization mode represents a key driver of phenotypic diversification at the cellular level, akin to morphological evolution at the organismal level in response to ecological niches. Yet while fertilization mode broadly impacts sperm component length evolution, these impacts vary depending on the sperm component considered and taxonomic level of analysis. Nevertheless, we suggest that evolutionary responses in sperm component lengths can be driven by a complex interaction between postcopulatory sexual selection, biophysical properties of the fertilization environment, and sperm-egg interactions that are not completely associated with fertilization mode. Unraveling how each of these factors, and their interactions, impact sperm component length evolution is necessary to understand the tremendous variation in sperm lengths across animals.

## Methods

### Sperm morphological database and classification of fertilization mode

Data from the SpermTree data repository^41^ were compiled, focusing only on species where sperm morphology consisted of a sperm head, midpiece, and a single flagellum (i.e., ‘standard’ morphology^41^). For 213 species, flagellum length was calculated by subtracting head length and midpiece length from the total length when it was clear in the original paper that this calculation would accurately reflect the length of the flagellum. Therefore, flagellum length reflects the part of the flagellum that does not include the midpiece (this sperm component is technically referred to as the principle piece, however, we use the more common term ‘flagellum’ hereafter). Flagellum length measurements includes the sperm endpiece, the short tip of the flagellum that contains only the axoneme, in species where the endpiece is present.

Data on sperm head, midpiece, and flagellum length for 1244 vertebrate species was present in the SpermTree repository. However, 141 species in the repository were not present in the phylogeny (described below), reducing the final sample size to 1103 species (Fig. 1a).

### Phylogenetic analyses

To account for shared evolutionary history, we used a phylogeny generated from the Open Tree of Life and time-calibrated using nodal dates from www.TimeTree.org. Details about the methods for tree construction can be found in Kahrl et al.^13^. All analyses were conducted using R version 4.1.1^71^ and all sperm lengths were natural log-transformed prior to analyses. Preliminary analyses demonstrated that an Ornstein-Uhlenbeck (OU) model of character evolution was the best fit all sperm components (Table S6). Therefore, all phylogenetic comparative methods were performed within an OU model framework. In analyses examining fertilization mode, analyses were performed across the full vertebrate dataset and within Osteichthyes and Amphibia, the two groups where there is within-clade variation in fertilization mode.

### Ancestral state reconstruction

To determine the number of transitions between fertilization modes we conducted stochastic character mapping of fertilization mode across our tree. We used the function *make.simmap* from the package *phytools* v. 0.7-80^72^ to reconstruct fertilization mode across the tree. We ran this function for 1000 iterations using two models, allowing all rates to differ (ARD) among fertilization modes, and compared this model to one where all rates were equal (ER) to produce 1000 stochastic character maps of each model (SIMMAPs). The output from these SIMMAPs was summarized using the function *describe.simmap* from the package *phytools* v. 0.7-80^72^ and loglikelihood values were used to determine the best fitting model.

### Phylogenetic linear models

Sperm component lengths were examined using phylogenetic linear models with an OU error structure using the ‘*phylolm’* function from the package *phylolm* v. 2.6.2^73^. To test the hypothesis that fertilization mode influences sperm component lengths, we compared sperm head, midpiece, and flagellum lengths between internal and external fertilizers.

### Evolutionary rates

Differences in evolutionary rates of sperm component lengths between fertilization modes were examined using the R package *OUwie* v. 2.1^42^. *OUwie* facilitates comparisons of several models of trait evolution in an OU framework. The parameters in these models include the evolutionary rate (σ^2^; an estimate of how rapidly a trait diversifies), the evolutionary optima (θ; the optimal size of a trait for one or more states, such as fertilization modes), and the selection parameter (α; an estimate of how strong trait values are pulled towards the evolutionary optima). These parameters are allowed to vary or are constrained in different models. To take into account the uncertainty of ancestral states of fertilization modes across the vertebrate tree, we first created a set of 20 SIMMAP trees using the function ‘*make.simmap’*. We used this initial set of 20 trees for model comparisons within *OUwie* (note that the number of trees used in this step of our analyses was small given the computationally expensive nature of these models). We then ran five different models, which vary in whether model parameters are constrained or not, to test whether evolutionary rates are the same or different among sperm components (details in Table S4). We used corrected Akaike information criterion scores and calculated Akaike weights to determine the best-supported model. After identifying the evolutionary model that best fit the data for each sperm component, we ran an additional 30 SIMMAPs using that model (giving a total of 50 independent model runs). We tested for differences in the estimates of the evolutionary parameters between fertilization modes using *t*-tests. This procedure was done for all species, and was repeated for Osteichthyes and Amphibia.

### Comparing rates of evolution between sperm components

To test for differences in the rates of evolution between the sperm component lengths we used the function ‘*mvOU’* in the package *mvMORPH* 1.1.4^43^. These models estimate the evolutionary optima (θ) either as single values for each trait or as multiple optima for different groups (in our case, fertilization modes) and evolutionary rates (σ^2^) are estimated as a matrix, in which the diagonal of the matrix is the observed trait rate, and the off-diagonals are the correlated rate estimates between traits. To determine whether rates of evolution differ between traits, we compared the fit of models that differ in (i) whether or not models include single- or multiple-optima estimates by fertilization mode, and (ii) whether or not the observed rate matrix or a modified rate matrix was a better fit for the model. The rate matrix was modified by either constraining the evolutionary rate (σ^2^) matrix (where off-diagonal correlated rates are set to 0) to model independent evolution of each sperm component, or by setting the rate values to be equal along the diagonal (evolutionary rate elements are equal, correlated rates are allowed to vary) to model equal rates of evolution for each sperm component. Therefore, we generated models that estimated the rate matrix of all three sperm components either as the observed values, the independent values, or equal values. Each of these three rate matrices were run with as either single- or multiple- optima models, thus resulting in six different models in our comparison. These variants of evolutionary models allowed us to test for the best fit model for all three sperm traits simultaneously, using corrected Akaike information criterion scores and Akaike weights to determine the best-supported model. If rates are different between sperm traits, the observed rates model or the independent rates model will be preferred over the equal rates model. We applied these models to the entire dataset of vertebrates, to all externally fertilizing vertebrates, to all internally fertilizing vertebrates, and finally to each class of vertebrates. When there was no variation in fertilization mode in a group, only single-optima models were run. We also ran post hoc tests using the same procedure to test for differences in the rates of evolution of all pairwise combinations of sperm traits (e.g., head and midpiece lengths, midpiece and flagellum lengths, and flagellum and head lengths).

### Reporting summary

Further information on research design is available in the Nature Portfolio Reporting Summary linked to this article.

## Supplementary information

Supplementary Information

Reporting Summary

## Data Availability

All data used in this study have been deposited in the Open Science Framework under accession code 5d72s: https://osf.io/5d72s. Source data are provided with this paper.

## Source data

Source Data

## Code availability

All code used in this study has been deposited in the Open Science Framework under accession code 5d72s: https://osf.io/5d72s.
