# Supplementary material for: Fertilization mode differentially impacts the evolution of vertebrate sperm components
Source: Nat Commun. 2022 Nov 10;13:6809. doi: 10.1038/s41467-022-34609-7 (PMC9649735; doi:10.1038/s41467-022-34609-7)
Supplement: Supplementary file 1 — Supplementary Information [file 41467_2022_34609_MOESM1_ESM.pdf]

Fertilization mode differentially impacts the evolution of vertebrate sperm components

Ariel F. Kahrl[1*], Rhonda R. Snook[1,2], and John L. Fitzpatrick[1,3]

Supplementary Material

Table S1. Vertebrate sperm component lengths. Sample size (N) and mean ± SE of sperm component (head, midpiece, and flagellum) lengths (μm) for vertebrates. Data are presented across all vertebrates in the full dataset (a) and divided by major taxonomic divisions among vertebrates (b-g). Across all vertebrates (a) and within Osteichthyes (c) and Amphibia (d) data are further divided based on fertilization mode (i.e. external vs internal fertilizers, indicated in italics).

**Taxonomic Level**	**N**	**Head Length**	**Midpiece Length**	**Flagellum Length**
a) Vertebrates	1103	12.6± 0.4	27.7± 1.4	53.9 ± 0.9
External fertilizers	191	9.6 ± 0.9	2.1 ± 0.2	38.6 ± 1.4
Internal fertilizers	912	13.3 ± 0.4	33.0 ± 1.6	57.1 ± 1.1
b) Chondrichthyes	51	43.1 ± 1.8	11.8 ± 0.6	91.2 ± 3.8
c) Osteichthyes	134	3.3 ± 0.5	1.5 ± 0.3	36.9 ± 1.7
External fertilizers	121	2.9 ± 0.5	1.0 ± 0.1	37.7 ± 1.8
Internal fertilizers	13	6.9 ± 1.5	6.8 ± 3.2	29.8 ± 2.6
d) Amphibia	104	26.2 ± 2.5	19.4 ± 5.9	56.7 ± 3.4
External fertilizers	70	21.1 ± 1.5	4.2 ± 0.6	40.2 ± 2.1
Internal fertilizers	34	36.6 ± 6.8	50.7 ± 17.2	90.7 ± 6.4
e) Reptilia	117	15.5 ± 0.5	20.0 ± 2.6	59.4 ± 1.9
f) Aves	237	14.2 ± 0.2	70.6 ± 4.5	27.7 ± 1.4
g) Mammalia	460	7.3 ± 0.2	18.8 ± 0.6	66.3 ± 1.5

Table S2. Comparison of ancestral character estimation models. Transition rates and the average number of shifts between fertilization modes are presented for two ancestral character estimation models, one where all rates were different (ARD, i.e., backwards and forwards rates between states are permitted to have different values), and one all rates were equal (ER). The maximum likelihood estimates (lnL) of the models were used to identify the model with the best fit to our data. Overall, we detected 13.14 shifts averaged across 1000 SIMMAPs in the ARD model, and 11.78 in the ER model.

SIMMAP model	lnL of model fit	Maximum likelihood estimates of the transition rates		Average number of shifts
All Rates Different	-49.02	External → Internal	1.18×10^{-3}	13.14
		Internal → External	~0	0
Equal Rates	-57.23	External → Internal	3.54×10^{-4}	8.49
		Internal → External	3.54×10^{-4}	3.28

Table S3. Fertilization mode and sperm component lengths. Phylogenetic linear models testing for differences in sperm component lengths between fertilization modes in all vertebrates, in Osteichthyes, and in Amphibia. Models were run using an OU model of evolution, with the alpha parameter defining the error structure in the models.

**Group, Trait**	**α**	***t***	***P***
Vertebrates			
Head Length	0.01	7.90	<0.001
Midpiece Length	0.03	13.99	<0.001
Flagellum Length	0.06	5.64	<0.001
Osteichthyes			
Head Length	0.01	2.92	0.004
Midpiece Length	0.04	6.11	<0.001
Flagellum Length	0.11	-1.71	0.089
Amphibia			
Head Length	0.01	0.84	0.40
Midpiece Length	0.02	4.10	<0.001
Flagellum Length	0.14	7.94	<0.001

Table S4. *OUwie* model fitting parameters. We compare five different models (OU1, OUM, OUMV, OUMA, OUMVA) that estimate the evolutionary parameters of sperm component evolution between fertilization modes using datasets that assessed (a) vertebrates, (b) Osteichthyes and (c) Amphibia present in the dataset. These models differ in the extent to which the evolutionary parameters are allowed to vary between fertilization modes or are held equal. The most basic model, OU1, holds all parameters (evolutionary optima (θ), evolutionary rate (σ^2), and selection parameter (α)) equal between fertilization modes. OUM allows for the evolutionary optima (θ) to vary, but the rates (σ^2) and alpha (α) are held equal. OUMV allows the evolutionary optima (θ) and rates (σ^2) to vary between fertilization modes, but holds alpha (α) equal. OUMA allows the evolutionary optima (θ) and alpha (α) to vary, but holds the rate (σ^2) equal between fertilization modes. Finally, OUMVA allows all parameters (θ, σ^2, α) to vary between fertilization modes. Therefore, only two models in this set (OUMV, and OUMVA) allow the evolutionary rate to vary between fertilization modes. All models are compared using the maximum likelihood estimates (lnL) and corrected Akaike Information Criterion (AICc) values presented. A value of Δ_I <2, the difference between the AICc values of the best fit and observed model, indicates equivalent models of evolution, and the Akaike weights (ω_i) indicate the strength of evidence for each model. The mean (and 95% CI) estimates of the evolutionary optima (θ), rate of evolution (σ^2), and selection parameter (α) for the best fit model (highlighted in grey). In models where the evolution parameters did not differ between fertilization modes, the shared parameter values are presented in an intermediate position between the columns for external and internal fertilization. The magnitude of the difference in evolutionary rate (i.e., evolutionary rate difference) is provided for each comparison of σ^2 between fertilization modes, with brackets indicating whether evolutionary rate values were greater in external (E) or internal (I) fertilizers.

Table S4a

Vertebrates	lnL	AICc	Δ_i	ω_i	LRT	*P*	Parameter	External Fertilization ± 95% CI	Internal Fertilization ± 95% CI	Evolutionary rate difference
Head Length										
OUMVA	-384.54	781.17	0	1	17.76	<0.001	θ	1.663 ± 0.074	3.279 ± 0.041	
OUMA	-393.42	796.89	15.72	<0.001			σ^2	0.013 ± 0.003	0.007 ± 0.002	x1.86 (E>I)
OUMV	-397.78	805.62	24.45	<0.001			α	0.007 ± 5.4e-4	0.011 ± 4.3e-4	
OUM	-398.05	804.22	23.05	<0.001						
OU1	-426.42	858.86	77.69	<0.001						
Midpiece Length										
OUMA	-886.51	1783.08	0	1	12.82	<0.001	θ	0.097 ± 0.001	2.366 ± 0.002	
OUMV	-892.92	1795.91	12.83	0.001			σ^2	0.042 ± 9.0e-4		NA (E=I)
OUMVA	-892.12	1796.24	13.16	0.001			α	0.027 ± 0.002	0.028 ± 0.002	
OUM	-895.56	1799.15	16.07	<0.001						
OU1	-968.07	1942.17	159.09	<0.001						
Flagellum Length										
OUMV	-497.49	1005.05	0	0.93	5.08	0.024	θ	3.609 ± 5.0e-4	3.985 ± 4.5e-4	
OUMA	-500.03	1010.12	5.07	0.073			σ^2	0.024 ± 6.9e-5	0.047 ± 1.1e-4	x1.96 (E<I)
OUM	-513.48	1035.01	29.96	<0.001			α	0.063 ± 3.0e-4		
OUMVA	-522.31	1056.70	51.65	<0.001						
OU1	-530.09	1066.21	61.16	<0.001						

Table S4b

Osteichthyes	lnL	AICc	Δ_i	ω_i	LRT	*P*	Parameter	External Fertilization ± 95% CI	Internal Fertilization ± 95% CI	Evolutionary rate difference
Head Length										
OUM	-90.71	189.73	0	0.61	0.54	0.46	θ	0.948 ± 0.007	2.048 ± 0.078	NA (E=I)
OUMV	-90.44	190.88	1.15	0.34			σ^2	0.015 ± 2.1e-4		
OU1	-94.36	194.91	5.18	0.05			α	0.015 ± 2.5e-4		
OUMA	-	-	-	-						
OUMVA	-	-	-	-						
Midpiece Length										
OUMV	-13.57	37.06	0	1	35.84	<0.001	θ	0.620 ± 0.002	1.694 ± 0.039	x12.57 (E<I)
OUM	-31.49	71.30	34.27	0			σ^2	0.007 ± 2.2e-4	0.088 ± 0.030	
OU1	-46.20	98.58	61.52	0			α	0.044 ± 0.002		
OUMVA	-	-	-	-						
OUMA	-	-	-	-						
Flagellum Length										
OUM	-52.98	114.27	0	0.50	0.60	0.44	θ	3.565 ± 0.001	3.356 ± 0.003	NA (E=I)
OU1	-52.68	115.50	1.23	0.27			σ^2	0.054 ± 3.0e-3		
OUMV	-54.66	115.85	1.58	0.23			α	0.188 ± 0.001		
OUMA	-	-	-	-						
OUMVA	-	-	-	-						

Table S4c

Amphibia	lnL	AICc	Δ_i	ω_i	LRT	*P*	Parameter	External Fertilization ± 95% CI	Internal Fertilization ± 95% CI	Evolutionary rate difference
Head Length										
OUMVA	-44.28	101.44	0	0.86	9.32	0.002	θ	3.031 ± 0.002	3.283 ± 0.060	
OUMA	-48.94	108.50	7.06	0.03			σ^2	0.004 ± 1.9e-3	0.111 ± 0.086	x27.75 (E<I)
OUMV	-49.50	109.62	8.17	0.01			α	0.035 ± 1.5e-3	0.008 ± 0.002	
OUM	-49.98	108.36	6.92	0.03						
OU1	-50.16	106.56	5.12	0.07						
Midpiece Length										
OUMVA	-87.62	188.11	0	1	31.88	<0.001	θ	1.139 ± 0.046	4.061 ± 0.761	
OUMV	-103.56	217.75	29.64	<0.001			σ^2	0.012 ± 6.6e-3	1.668 ± 1.747	X139 (E<I)
OUM	-106.85	222.10	33.99	<0.001			α	0.057 ± 5.4e-3	0.025 ± 0.046	
OU1	-112.51	231.26	43.15	<0.001						
OUMA	-	-	-	-						
Flagellum Length										
OUMV	-30.39	71.40	0	0.81	5.18	0.022	θ	3.681 ± 2.2e-4	4.423 ± 1.2e-3	
OUM	-32.98	74.36	2.96	0.18			σ^2	0.036 ± 1.1e-4	0.069 ± 3.3e-4	x1.92 (E<I)
OU1	-51.37	108.97	37.57	<0.001			α	0.167 ± 7.7e-4		
OUMA	-	-	-	-						
OUMVA	-	-	-	-						

Supplementary Table S5. mvMORPH model support and parameter estimates for sperm morphological evolution across all vertebrates in our dataset, internal and external fertilizers, and each vertebrate class. Models were built allowing single or multiple evolutionary (θ) optima for internal and external fertilizers (listed as single- or multi-optima). In addition to allowing the optima to vary, the rate matrix (σ) was either allowed to be unconstrained (observed rates), the rates had no covariance (rates independent), or rates were held equal for all traits (rates equal). Therefore, in groups with both internal and external fertilizers six different model types were present, to allow the rate matrix to vary, for rates to be independent, or for rates to be held equal in both single- and multi-optima models. All the models were tested fitting an Ornstein-Uhlenbeck (OU) process and compared using corrected Akaike Information criterion (AICc). The evolutionary parameter estimates (on the right side of the table) are presented from the best fit model (highlighted in grey), which represent the evolutionary optima (θ) for each sperm component and for each fertilization mode (where relevant), as well as the evolutionary selection variance-covariance (α) matrix and the evolutionary rate variance-covariance (σ) matrix. The α matrix estimates are included in these model out-puts, but are not interpreted in our findings. These models were run for all traits, and for all pairwise combinations of traits in post-hoc tests.

Supplementary Table S5.

Model	AICc	ΔAICc	ω_i	Evolutionary Parameter Estimates<br>Parameter		Head	Midpiece	Flagellum
**a) Vertebrates**								
Multi-optima, observed rates	3271.6	0	1	θ	**External**	1.486	0.122	3.628
Single-optima, observed rates	3456.6	185.0	0		**Internal**	3.012	2.370	4.066
Multi-optima, independent rates	3492.1	220.5	0	α	**Head**	0.015	-0.002	-0.007
Multi-optima, equal rates	3583.7	312.1	0		**Midpiece**	-0.002	0.032	0.015
Single-optima, independent rates	3661.5	389.9	0		**Flagellum**	-0.007	0.015	0.076
Single-optima, equal rates	3752.0	480.4	0	σ	**Head**	0.019	0.014	0.010
					**Midpiece**	0.014	0.062	0.010
					**Flagellum**	0.010	0.010	0.044
**b) Externally fertilizing vertebrates**								
Single-optima, observed rates	631.5	0	1	θ		1.594	0.232	3.619
Single-optima, independent rates	641.2	9.7	0	α	**Head**	0.026	-0.030	-0.016
Single-optima, equal rates	675.4	43.9	0		**Midpiece**	-0.030	0.055	0.005
					**Flagellum**	-0.015	0.005	0.145
				σ	**Head**	0.015	-0.006	0.007
					**Midpiece**	-0.006	0.050	0.002
					**Flagellum**	0.007	0.002	0.037
**c) Internally fertilizing vertebrates**								
Single-optima, observed rates	2372.1	0	1	θ		2.848	2.335	4.059
Single-optima, equal rates	2757.1	207.0	0	α	**Head**	0.018	0.005	-0.001
Single-optima, independent rates	2640.8	269.7	0		**Midpiece**	0.005	0.035	0.021
					**Flagellum**	-0.001	0.021	0.077
				σ	**Head**	0.022	0.018	0.014
					**Midpiece**	0.018	0.058	0.013
					**Flagellum**	0.014	0.013	0.048

Supplementary Table S5., continued

Model	AICc	ΔAICc	ω_i	Evolutionary Parameter Estimates				
				Parameter		Head	Midpiece	Flagellum
**d) Elasmobranchii**								
Single-optima, observed rates	68.3	0	0.93	θ		3.781	2.464	4.474
Single-optima, equal rates	73.4	5.14	0.07	α	**Head**	0.037	-0.032	0.015
Single-optima, independent rates	84.6	15.33	0		**Midpiece**	-0.032	0.051	-0.021
					**Flagellum**	0.015	-0.021	0.019
				σ	**Head**	0.005	-0.003	0.001
					**Midpiece**	-0.003	0.009	-0.002
					**Flagellum**	0.001	-0.001	0.002
**e) Osteichthyes**								
OU Multi-optima, independent rates	533.1	0	1	θ	**External**	0.933	-0.198	3.594
OU Multi-optima, observed rates	536.3	3.2	0		**Internal**	1.970	1.583	3.385
OU Multi-optima, equal rates	540.8	7.7	0	α	**Head**	0.030	-0.012	-0.034
OU Single-optima, independent rates	559.6	26.5	0		**Midpiece**	-0.012	0.036	-0.002
OU Single-optima, observed rates	562.1	29.0	0		**Flagellum**	-0.033	-0.002	0.197
OU Single-optima, equal rates	566.5	33.3	0	σ	**Head**	0.018	0	0
					**Midpiece**	0	0.037	0
					**Flagellum**	0	0	0.051
**f) Externally fertilizing Osteichthyes**								
OU Single-optima, independent rates	436.9	0	0.68	θ		0.944	-0.206	3.604
OU Single-optima, observed rates	438.5	1.6	0.31	α	**Head**	0.037	-0.014	-0.052
OU Single-optima, equal rates	446.0	9.1	0.01		**Midpiece**	-0.014	0.053	-0.004
					**Flagellum**	-0.052	-0.004	0.203
				σ	**Head**	0.016	0	0
					**Midpiece**	0	0.042	0
					**Flagellum**	0	0	0.049

Supplementary Table S5., continued

Model	AICc	ΔAICc	ω_i	Evolutionary Parameter Estimates: Parameter		Head	Midpiece	Flagellum
**g) Internally fertilizing Osteichthyes**								
OU Single-optima, equal rates	81.9	0	0.99	θ		1.656	1.151	3.338
OU Single-optima, independent rates	91.4	9.5	0.01	α	**Head**	1.054	-0.028	2.214
OU Single-optima, observed rates	118.6	36.7	0		**Midpiece**	-0.028	0.107	-0.276
					**Flagellum**	2.214	-0.276	4.156
				σ	**Head**	0.277	0.244	0.166
					**Midpiece**	0.244	0.277	0.042
					**Flagellum**	0.166	0.042	0.277
**h) Amphibia**								
OU Multi-optima, equal rates	350.59	0	0.75	θ	**External**	2.907	0.975	3.694
OU Multi-optima, observed rates	353.82	3.23	0.15		**Internal**	3.328	2.938	4.405
OU Multi-optima, independent rates	354.76	4.17	0.10	α	**Head**	0.099	-0.048	0.047
OU Single-optima, equal rates	391.80	41.21	0		**Midpiece**	-0.048	0.035	-0.019
OU Single-optima, observed rates	395.90	45.31	0		**Flagellum**	0.047	-0.019	0.115
OU Single-optima, independent rates	419.63	69.04	0	σ	**Head**	0.040	-0.007	0.029
					**Midpiece**	-0.007	0.040	0.006
					**Flagellum**	0.029	-0.006	0.040
**i) Externally fertilizing Amphibia**								
OU Single-optima, independent rates	136.5	0	0.92	θ		3.009	1.134	3.682
OU Single-optima, observed rates	142.1	5.6	0.05	α	**Head**	0.204	-0.043	-0.245
OU Single-optima, equal rates	143.6	7.1	0.03		**Midpiece**	-0.043	0.083	0.027
					**Flagellum**	-0.245	0.027	0.454
				σ	**Head**	0.018	0	0
					**Midpiece**	0	0.081	0
					**Flagellum**	0	0	0.049

Supplementary Table S5., continued

Model	AICc	ΔAICc	ω_i	Evolutionary Parameter Estimates<br>Parameter		Head	Midpiece	Flagellum
**j) Internally fertilizing Amphibia**								
OU Single-optima, independent rates	171.9	0	0.76	θ		3.403	3.102	4.411
OU Single-optima, observed rates	174.4	2.4	0.23	α	**Head**	0.054	-0.029	-0.075
OU Single-optima, equal rates	182.6	10.6	0.01		**Midpiece**	-0.029	0.019	0.062
					**Flagellum**	-0.075	0.062	1.145
				σ	**Head**	0.016	0	0
					**Midpiece**	0	0.009	0
					**Flagellum**	0	0	0.422
**k) Reptilia**								
OU Single-optima, observed rates	-97.8	0	0.91	θ		2.753	1.864	4.047
OU Single-optima, equal rates	-93.1	4.7	0.09	α	**Head**	0.063	0.015	0.021
OU Single-optima, independent rates	-36.9	60.9	0		**Midpiece**	0.015	0.013	0.021
					**Flagellum**	0.021	0.021	0.066
				σ	**Head**	0.008	0.004	0.005
					**Midpiece**	0.004	0.011	0.005
**l) Aves**								
OU Single-optima, observed rates	255.8	0	1	θ		2.618	2.235	3.557
OU Single-optima, independent rates	325.4	69.6	0	α	**Head**	0.194	-0.002	-0.001
OU Single-optima, equal rates	329.9	74.1	0		**Midpiece**	-0.002	0.034	0.020
					**Flagellum**	-0.001	0.020	0.086
				σ	**Head**	0.018	0.021	-0.002
					**Midpiece**	0.021	0.103	-0.031
					**Flagellum**	-0.002	-0.031	0.043
**m) Mammalia**								
OU Single-optima, observed rates	802.3	0	0.89	θ		1.910	2.512	4.015
OU Single-optima, equal rates	806.7	4.4	0.11	α	**Head**	0.155	-0.011	0.044
OU Single-optima, independent rates	914.9	112.6	0		**Midpiece**	-0.010	0.083	-0.019
					**Flagellum**	0.044	-0.019	0.200
				σ	**Head**	0.051	0.027	0.035
					**Midpiece**	0.027	0.071	0.035
					**Flagellum**	0.035	0.035	0.084

Table S6. Comparison of parameters and fit for sperm component lengths under Brownian Motion, Ornstein-Uhlenbeck and Early Burst evolutionary models. We compared the fit of Brownian Motion (BM), Ornstein-Uhlenbeck (OU), and Early-Burst (EB) models of character evolution for a) sperm head length, b) sperm midpiece length, and c) sperm flagellum length using the function *fitContinuous* from the package *geiger* v. 2.0.6 (Harmon et al. 2008). The Brownian rate parameter, σ^2, selection strength parameter, α, and rate of evolutionary change parameter, *a*, are presented for the Brownian Motion, Ornstein-Uhlenbeck and Early Burst models, respectively. Models were compared using the maximum likelihood estimates (lnL) and corrected Akaike Information Criterion (AICc) values presented. A value of Δ_I <2, the difference between the AICc values of the best fit and observed model, indicates equivalent models of evolution, and the Akaike weights (ω_i) indicate the strength of evidence for each model.

Model	Parameters	lnL	AICc	Δ_i	ω_i
**a) Sperm head length**					
Brownian Motion	$\sigma^2 = 1.56 \times 10^{-2}$	-496.96	997.93	139	0
Ornstein-Uhlenbeck	$\alpha = 7.61 \times 10^{-3}$	-426.42	858.88	0	1
Early Burst	$a = 1.00 \times 10^{-6}$	-496.97	999.96	141	0
**b) Sperm midpiece length**					
Brownian Motion	$\sigma^2 = 4.44 \times 10^{-2}$	-1071.46	2146.95	205	0
Ornstein-Uhlenbeck	$\alpha = 1.35 \times 10^{-2}$	-968.07	1942.16	0	1
Early Burst	$a = 1.00 \times 10^{-6}$	-1071.47	2148.97	207	0
**c) Sperm flagellum length**					
Brownian Motion	$\sigma^2 = 2.82 \times 10^{-2}$	-821.72	1647.74	581	0
Ornstein-Uhlenbeck	$\alpha = 6.13 \times 10^{-2}$	-530.09	1066.21	0	1
Early Burst	$a = 1.00 \times 10^{-6}$	-821.73	1649.48	583	0
