# Supplementary material for: Fertilization mode differentially impacts the evolution of vertebrate sperm components
Source: Nat Commun. 2022 Nov 10;13:6809. doi: 10.1038/s41467-022-34609-7 (PMC9649735; doi:10.1038/s41467-022-34609-7)
Supplement: Supplementary file 2 — Reporting Summary [file 41467_2022_34609_MOESM2_ESM.pdf]

nature portfolio

Corresponding author(s): Ariel Kahrl, NCOMMS-22-18061-T

Last updated by author(s): 10/26/2022

Reporting Summary

Nature Portfolio wishes to improve the reproducibility of the work that we publish. This form provides structure for consistency and transparency in reporting. For further information on Nature Portfolio policies, see our Editorial Policies and the Editorial Policy Checklist.

Statistics

For all statistical analyses, confirm that the following items are present in the figure legend, table legend, main text, or Methods section.

n/a	Confirmed	
☐	☒	The exact sample size (*n*) for each experimental group/condition, given as a discrete number and unit of measurement
☐	☒	A statement on whether measurements were taken from distinct samples or whether the same sample was measured repeatedly
☐	☒	The statistical test(s) used AND whether they are one- or two-sided<br>*Only common tests should be described solely by name; describe more complex techniques in the Methods section.*
☐	☒	A description of all covariates tested
☐	☒	A description of any assumptions or corrections, such as tests of normality and adjustment for multiple comparisons
☐	☒	A full description of the statistical parameters including central tendency (e.g. means) or other basic estimates (e.g. regression coefficient) AND variation (e.g. standard deviation) or associated estimates of uncertainty (e.g. confidence intervals)
☐	☒	For null hypothesis testing, the test statistic (e.g. *F*, *t*, *r*) with confidence intervals, effect sizes, degrees of freedom and *P* value noted<br>*Give P values as exact values whenever suitable.*
☒	☐	For Bayesian analysis, information on the choice of priors and Markov chain Monte Carlo settings
☒	☐	For hierarchical and complex designs, identification of the appropriate level for tests and full reporting of outcomes
☒	☐	Estimates of effect sizes (e.g. Cohen's *d*, Pearson's *r*), indicating how they were calculated

Our web collection on statistics for biologists contains articles on many of the points above.

Software and code

Policy information about availability of computer code

Data collection	*Provide a description of all commercial, open source and custom code used to collect the data in this study, specifying the version used OR state that no software was used.*
Data analysis	Data for this study was analyzed using the following R software packages: phytools v 0.7-80, phylolm v 2.6.2, OUwie v 2.1, mvMORPH v 1.1.4.<br><br>All code used in this study is publicly available on the Open Science Framework at https://osf.io/5d72s.

For manuscripts utilizing custom algorithms or software that are central to the research but not yet described in published literature, software must be made available to editors and reviewers. We strongly encourage code deposition in a community repository (e.g. GitHub). See the Nature Portfolio guidelines for submitting code & software for further information.

Data

Policy information about availability of data

All manuscripts must include a data availability statement. This statement should provide the following information, where applicable:

- Accession codes, unique identifiers, or web links for publicly available datasets
- A description of any restrictions on data availability
- For clinical datasets or third party data, please ensure that the statement adheres to our policy

Data used in this study was originally collected from SpermTree which is available at: https://spermtree.org/.
All data used in this study are publicly available on the Open Science Framework at https://osf.io/5d72s.

Human research participants

Policy information about studies involving human research participants and Sex and Gender in Research.

Reporting on sex and gender	NA
Population characteristics	NA
Recruitment	NA
Ethics oversight	NA

Note that full information on the approval of the study protocol must also be provided in the manuscript.

Field-specific reporting

Please select the one below that is the best fit for your research. If you are not sure, read the appropriate sections before making your selection.

☐ Life sciences ☐ Behavioural & social sciences ☒ Ecological, evolutionary & environmental sciences

For a reference copy of the document with all sections, see nature.com/documents/nr-reporting-summary-flat.pdf

Ecological, evolutionary & environmental sciences study design

All studies must disclose on these points even when the disclosure is negative.

Study description	We tested the hypothesis that the evolution of sperm morphology is shaped by fertilization mode. We used previously collected data on fertilization mode (internal and external) and sperm head, midpiece, and flagellum length for 1103 species of vertebrates. Using phylogenetic comparative methods we tested for differences in the length of each sperm component between fertilization modes, and for differences in the rates of evolution of each sperm component between fertilization modes as well as differences in the rates of evolution between each sperm component.
Research sample	We collected our data from the database SpermTree (Fitzpatrick et al. 2021). We chose to focus on the vertebrates in this database because they exhibit variation in fertilization modes both within and among clades, and have ample data to test our hypotheses.
Sampling strategy	Sample size was determined by the number of publications that had complete data in the SpermTree database.
Data collection	The data used in these analyses was procured from the SpermTree database.
Timing and spatial scale	Data were updated on SpermTree Jan 21, 2021. We used the data that were part of the most recent update.
Data exclusions	Incomplete data and species of invertebrates were excluded from these analyses.
Reproducibility	All data and code provided should ensure reproducibility of our findings.
Randomization	No randomization or covariates were necessary in this study.
Blinding	NA

Did the study involve field work? ☐ Yes ☒ No

Reporting for specific materials, systems and methods

We require information from authors about some types of materials, experimental systems and methods used in many studies. Here, indicate whether each material, system or method listed is relevant to your study. If you are not sure if a list item applies to your research, read the appropriate section before selecting a response.

Materials & experimental systems

n/a	Involved in the study
☒	☐ Antibodies
☒	☐ Eukaryotic cell lines
☒	☐ Palaeontology and archaeology
☒	☐ Animals and other organisms
☒	☐ Clinical data
☒	☐ Dual use research of concern

Methods

n/a	Involved in the study
☒	☐ ChIP-seq
☒	☐ Flow cytometry
☒	☐ MRI-based neuroimaging
